# Post-operative Complication at the Donor Site of Fibular Free Flap in a Pediatric Patient

**DOI:** 10.7759/cureus.21182

**Published:** 2022-01-12

**Authors:** Giri Valandhan Vedha, Sreejee Gopalakrishnan, Sathish J Kumar, Gopinath P Menon

**Affiliations:** 1 Oral and Maxillofacial Surgery, Sri Ramachandra Institute of Higher Education and Research, Chennai, IND; 2 Department of Plastic and Reconstructive Surgery, Sri Ramachandra Institute of Higher Education and Research, Chennai, IND; 3 Department of Orthopedic Surgery, Sri Ramachandra Institute of Higher Education and Research, Chennai, IND

**Keywords:** pediatric surgery, ankle valgus deformity, mandibular pathology, mandibular reconstruction, late donor-site complication

## Abstract

The fibular free flap is most frequently used for reconstructing the mandible owing to the adequate length of the bone with a reliable blood supply. It has a long vascular pedicle with adequate vessel diameter for anastomosis and also a good amount of skin paddle for coverage. However, the reports of post-operative complications at the donor site among pediatric patients are scarce in the literature. We present a pediatric case of ankle valgus deformity following vascularized fibular harvest.

## Introduction

Pediatric mandibular defects can have various causes. As a result, reconstruction of the pediatric mandible is challenging [1]. Fibular free flaps have been used to treat various postsurgical defects [2]. van Gemert JT et al. [3] reported major recipient-site complications such as arterial insufficiency/venous congestion, hemorrhage/hematoma, skin island necrosis, intra- and extraoral wound dehiscence, infection, plate fracture, osteonecrosis, and nonunion. Although donor-site morbidity is rare, it remains a significant concern with a fibular free flap [3]. This case report is of a 12-year-old boy with an ankle valgus deformity following a fibular free flap.

## Case presentation

**Figure 1:**
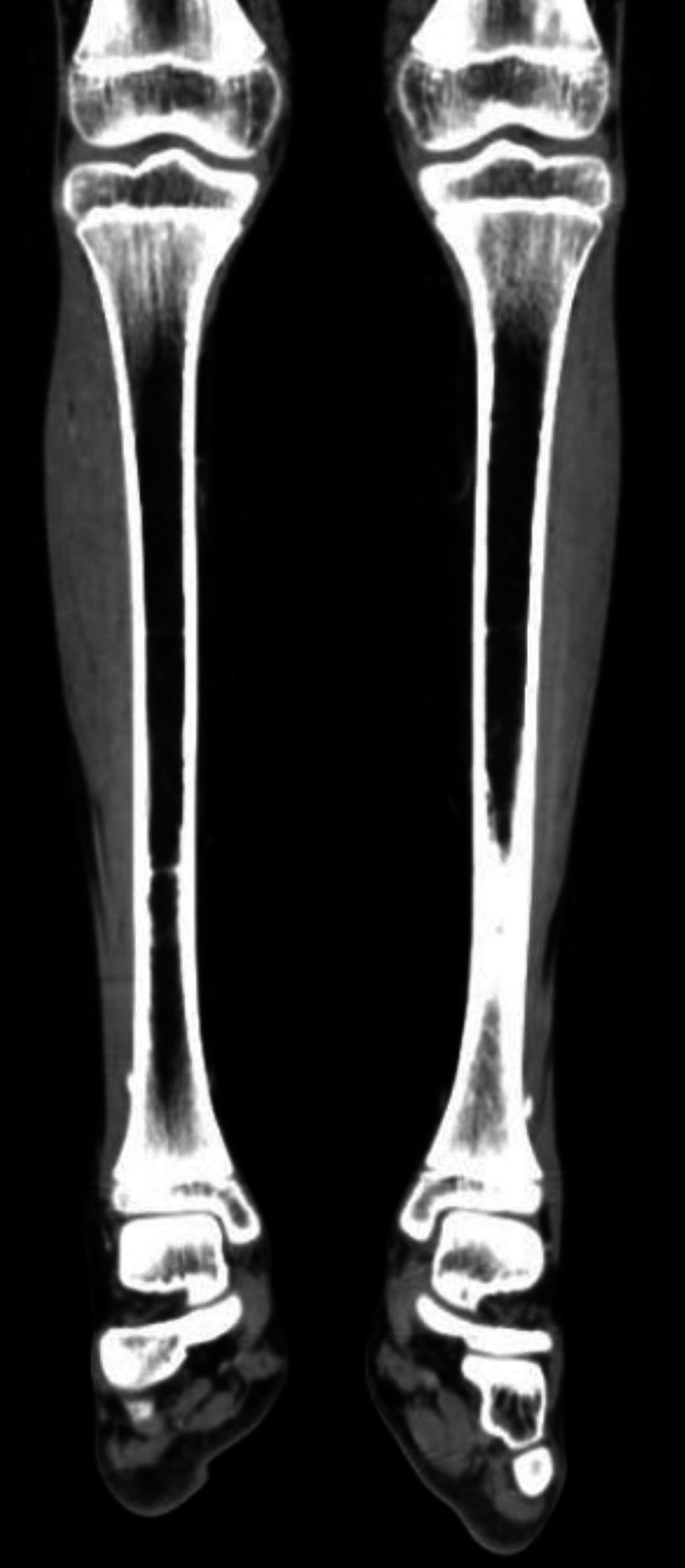
CT lower limb prior to fibula flap harvest, showing normal subtalar joint angulation.

A 12-year-old boy came to the oral and maxillofacial surgery department of our institution in 2017 with a chief complaint of swelling in the left lower jaw. A mandible CT scan revealed an expansile lytic lesion with a soap bubble appearance. Incisional biopsy was suggestive of acanthomatous ameloblastoma. A left hemimandibulectomy and reconstruction with a fibular free flap were planned. A lower limb CT arteriogram showed normal-appearing peroneal and tibial arteries (Figure 1).

**Figure 2:**
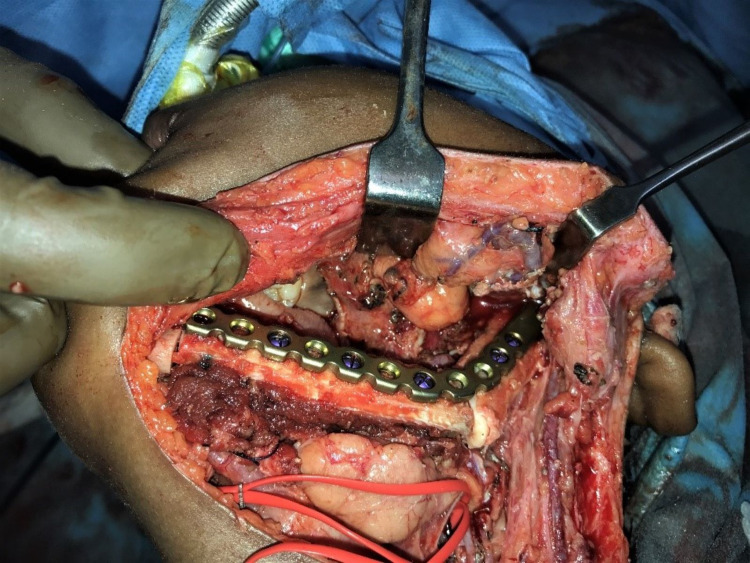
Intraoperative reconstruction of the left mandible with fibular free flap.

The planned procedures were performed with no complications (Figure 2).

**Figure 3:**
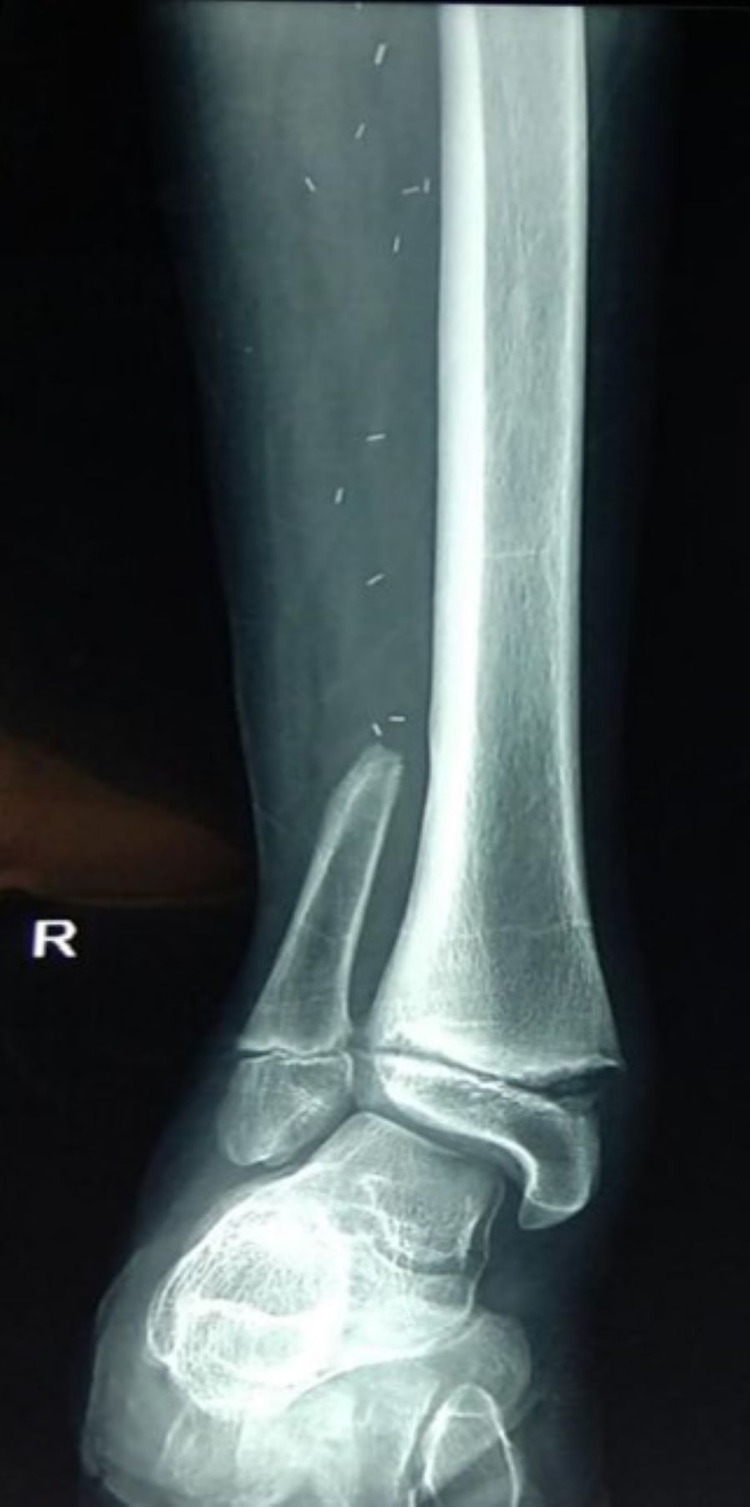
Anteroposterior radiograph of right ankle joint after fibula flap harvest, showing increased subtalar joint angulation.

The patient underwent appropriate post-operative rehabilitation. After 1.5 years, he started walking with a deformity. This deformity was progressive and impeded his gait. On examination, a valgus deformity of the right ankle was evident with valgus angulation of 15° (Figure 3).

**Figure 4:**
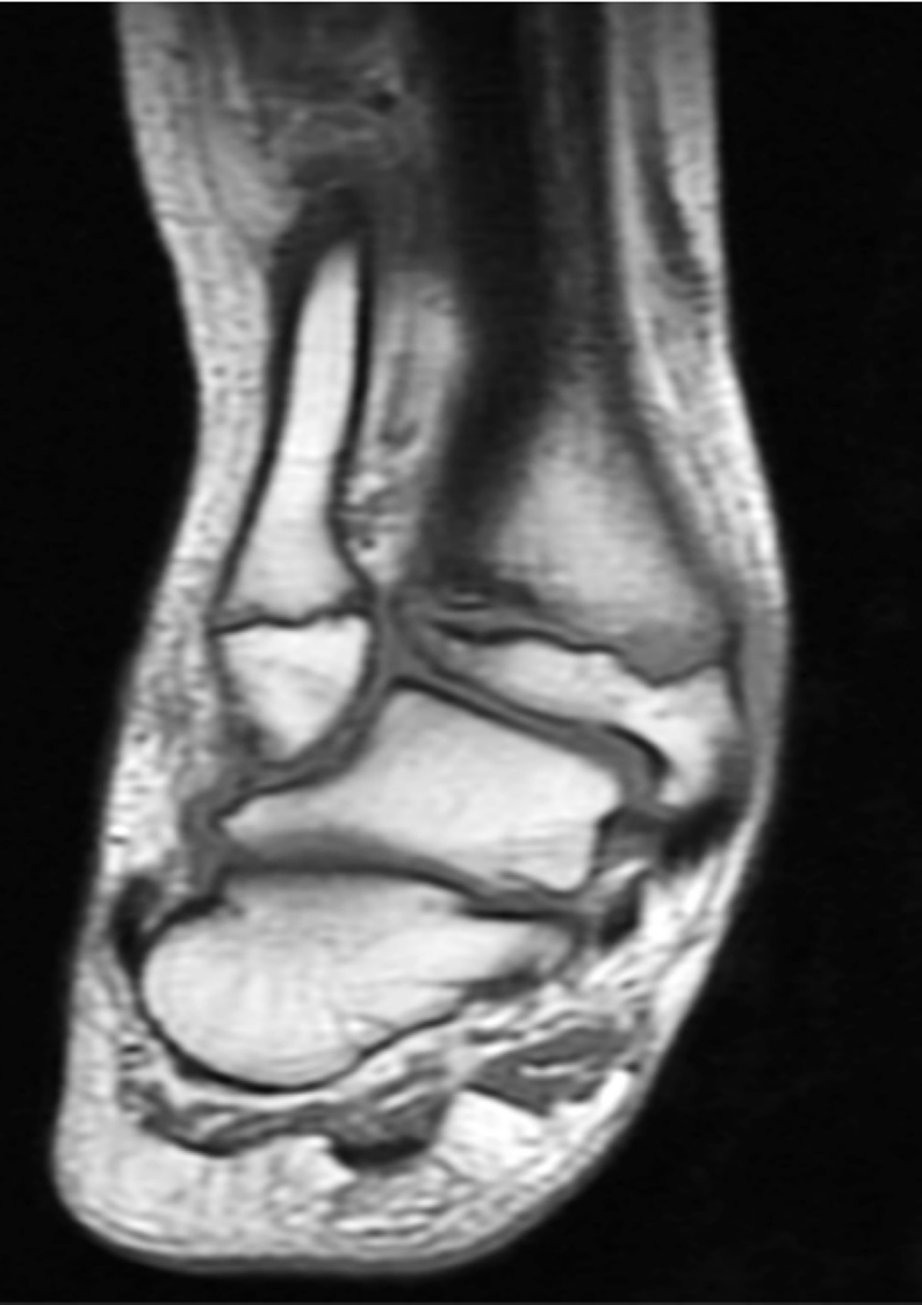
MRI of right ankle joint (coronal section) showing ankle valgus deformity.

The range of movement in the right ankle was full and free. MRI of the right subtalar joint done in May 2019 revealed an ankle valgus deformity of the subtalar joint (Figure 4) with characteristic features: (1) mild joint effusion in the ankle and subtalar region; (2) marrow edema involving the paraphyseal regions of the distal tibia and lateral malleolus; (3) irregularity with marrow edema of the metaphyseal end of the distal tibial epiphyseal plate (reactive changes due to the valgus deformity); and (4) a tear of the anterior talofibular ligament.

**Figure 5:**
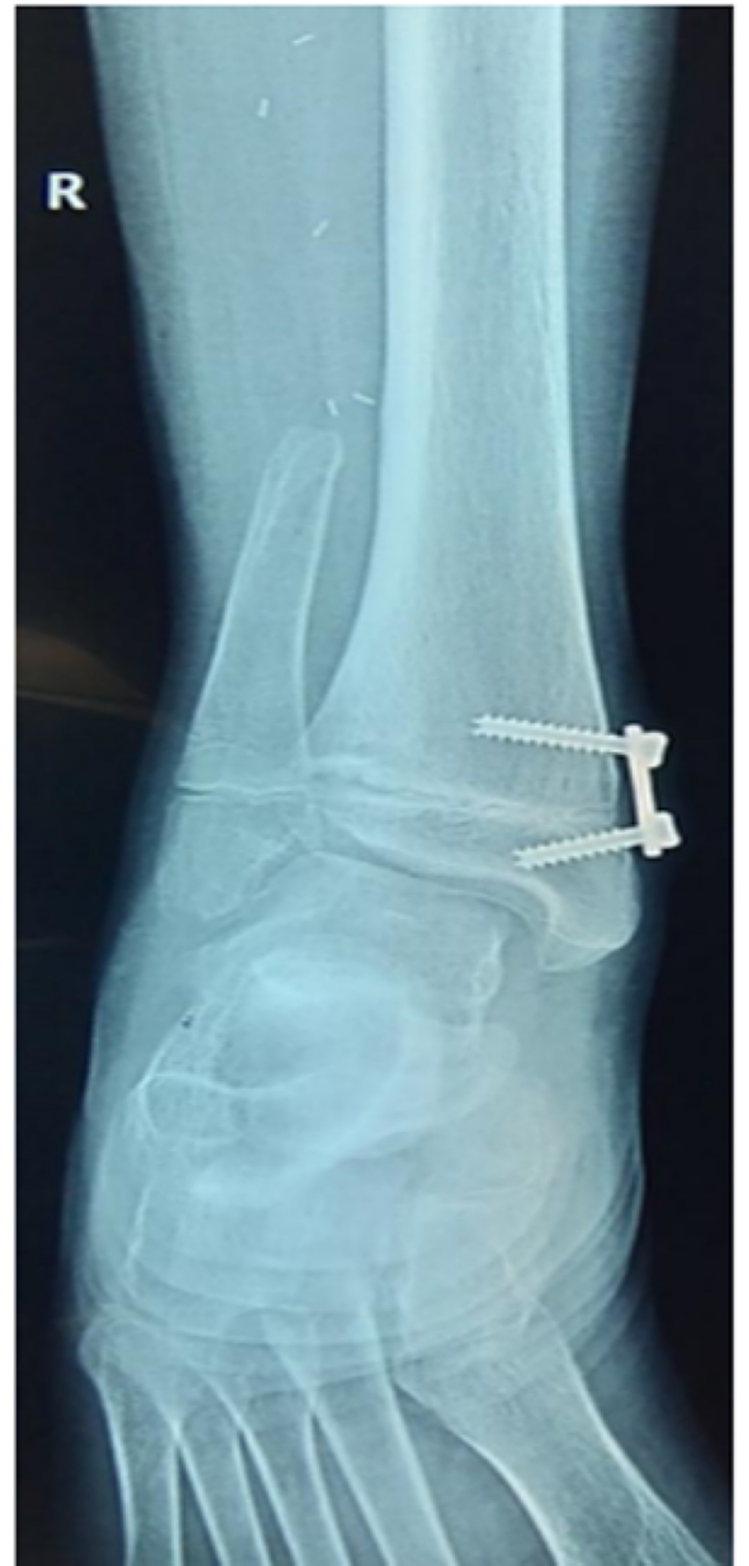
Anteroposterior radiograph of right ankle joint eight months after plating for growth modulation.

The patient underwent growth modulation surgery of the right ankle in June 2019 (Figure 5). Knee, ankle, and toe mobilization exercises were started postoperatively.

## Discussion

Several types of free flaps have been used in mandibular reconstruction. However, the fibula free flap has several advantages that make it an attractive option for reconstruction. These include consistent vascular anatomy, ease of harvest, significant bone stock, numerous endosteal and periosteal blood supplies that allow osteotomies, and the ability to accept dental implants [4,5].

Donor-site morbidity resulting from fibula free flap harvest is more common in children [6]. Children and adolescents who are obese suffer many orthopedic complications [7]. The fibula bears about 6%-16% of the load applied on the leg during weight-bearing [8,9]. This percentage of load exerted can change by the ankle's position [8,9]. Valgus deformities are due to proximal migration of the distal fibular segment that results in a progressive lateral shift of the talus, causing increased stress on the distal tibial growth plate during weight-bearing. Radiographic features include proximal fibular migration, lateral tibial epiphyseal atrophy, anteromedial fibular physeal arrest, and subluxation of the talus. Following fibular harvest, the incidence of valgus deformity in children is about 40% [1]. An age-residual fibula index (age in years + length of the residual fibula in cm) <16 is predictive of valgus deformity [1,6].

In a rabbit model, the tibial stress fractures were demonstrated and studied histologically by Li GP et al. [8]. The analysis revealed disruption of the cortical microvasculature that weakened the bone [8].

Ankle valgus deformity prevention involves stabilizing the distal fibula using a quadricortical, syndesmotic screw alone or combined with a tibial strut during flap harvest [8,9]. Unfortunately, the fibula flap harvest in children requires long-term follow-up until skeletal maturity to monitor ankle valgus deformity. This potential donor-site complication can be prevented by using a proper flap harvesting technique and aggressive physical rehabilitation.

## Conclusions

In children, mandibular reconstruction using a vascularized fibula free flap is a reliable procedure with excellent aesthetic and functional outcomes. However, donor-site morbidities are not uncommon, which should be acknowledged. Ankle valgus deformity is a potentially preventable devastating donor-site complication that warrants more attention among maxillofacial surgeons performing complex reconstructive procedures.
